# Genome assembly resources of genitourinary cancers for chromosomal aberration at the single nucleotide level

**DOI:** 10.1038/s41597-025-04801-7

**Published:** 2025-04-01

**Authors:** Hyunho Han, Hyung Ho Lee, Min Gyu Kim, Yoo Sub Shin, Jin Soo Chung, Jun Kim

**Affiliations:** 1 Department of Urology, Urological Science Institute, Yonsei University College of Medicine, Seoul, Republic of Korea; 2 Center for Urologic Cancer, National Cancer Center, Goyang, Republic of Korea; 3 Department of Convergent Bioscience and Informatics, College of Bioscience and Biotechnology, Chungnam National University, Daejeon, 34134 Korea

**Keywords:** Prostate cancer, Cancer genomics

## Abstract

Traditionally, the evolutionary perspective of cancer has been understood as gradual alterations in passenger/driver genes that lead to branching phylogeny. However, in cases of prostate adenocarcinoma and kidney renal cell carcinoma, macroevolutionary landmarks like chromoplexy and chromothripsis are frequently observed. Unfortunately, short-read sequencing techniques often miss these significant macroevolutionary changes, which involve multiple translocations and deletions at the chromosomal level. To resolve such genomic dark matters, we provided high-fidelity long-read sequencing data (78–92 Gb of ~Q30 reads) of six genitourinary tumour cell lines (one benign kidney tumour and two kidney and three prostate cancers). Based on these data, we obtained 12 high-quality, partially phased genome assemblies (Contig N50 1.85–29.01 Mb; longest contig 2.02–171.62 Mb), graph-based pan-genome variant sets (11.57 M variants including 60 K structural variants), and 5-methylcytosine sites (14.68%–27.05% of the CpG sites). We also identified several severe chromosome aberration events, which would result from chromosome break and fusion events. Our cancer genome assemblies will provide unprecedented resolution to understand cancer genome instability and chromosomal aberration.

## Background & Summary

Prostate cancer (PC) and renal cell carcinoma (RCC) are significant health concerns worldwide, accounting for 7.3% and 2.2% of cancer incidences, respectively^1^. For Both PC and RCC, researchers have focused on distinguishing these cancers at the genomic level, aiming to discern the “aggressive variants” from their slower-progressing counterparts. Central to their findings was the realization that chromosomal aberrations played a pivotal role in their malignant transformation and progression^2^.

In PC, TMPRSS2-ERG fusion (21q), recognized as the most prevalent genomic mutation, often advances through a sequential deletion of tumour suppressor genes (TSGs) including TP53, CDKN1B, and PTEN. This specific PC subtype’s transformation is predominantly governed by chromoplexy. This event involves multiple chromosomal translocations, leading to TSG deletions^3^.

In contrast, clear cell RCCs predominantly showcase a loss of the short arm of chromosome 3, evident in nearly 90% of cases. This is a classic instance of chromothripsis, a phenomenon defined by the multiple, simultaneous breakages of chromosomes. This is followed by a random rejoining of fragments post-repair, resulting in a cascade of gene deletions^4^. Given that several key TSGs (VHL, PBRM1, BAP1, SETD2) are located at 3p, its loss leads to a Loss of Heterozygosity (LOH) for these genes^5^.

Cancer genome assemblies are required for deciphering such chromosomal aberration at the single nucleotide level. Current methodologies for chromosome aberration are powerful, but typically depend on read-level analyses, of which lengths are too short to cover the whole structure of aberrant chromosomes^6–8^. Genome assembly based on long-read sequencing may resolve this problem, as it can provide highly contiguous and accurate DNA sequences of aberrant chromosomes^9–14^. However, cancer genome assembly has been very challenging and has suffered from cellular heterogeneity of the tumour tissue. This is because the genome assembly process needs a high amount of sequencing data (~20 × per genome) and its algorithms are typically designed for the homogenous diploid genome^15–24^. Thus, strategies to minimize cellular heterogeneity are demanded, and the use of cancer cell lines could be the easiest way^10,11^.

**Fig. 1:**
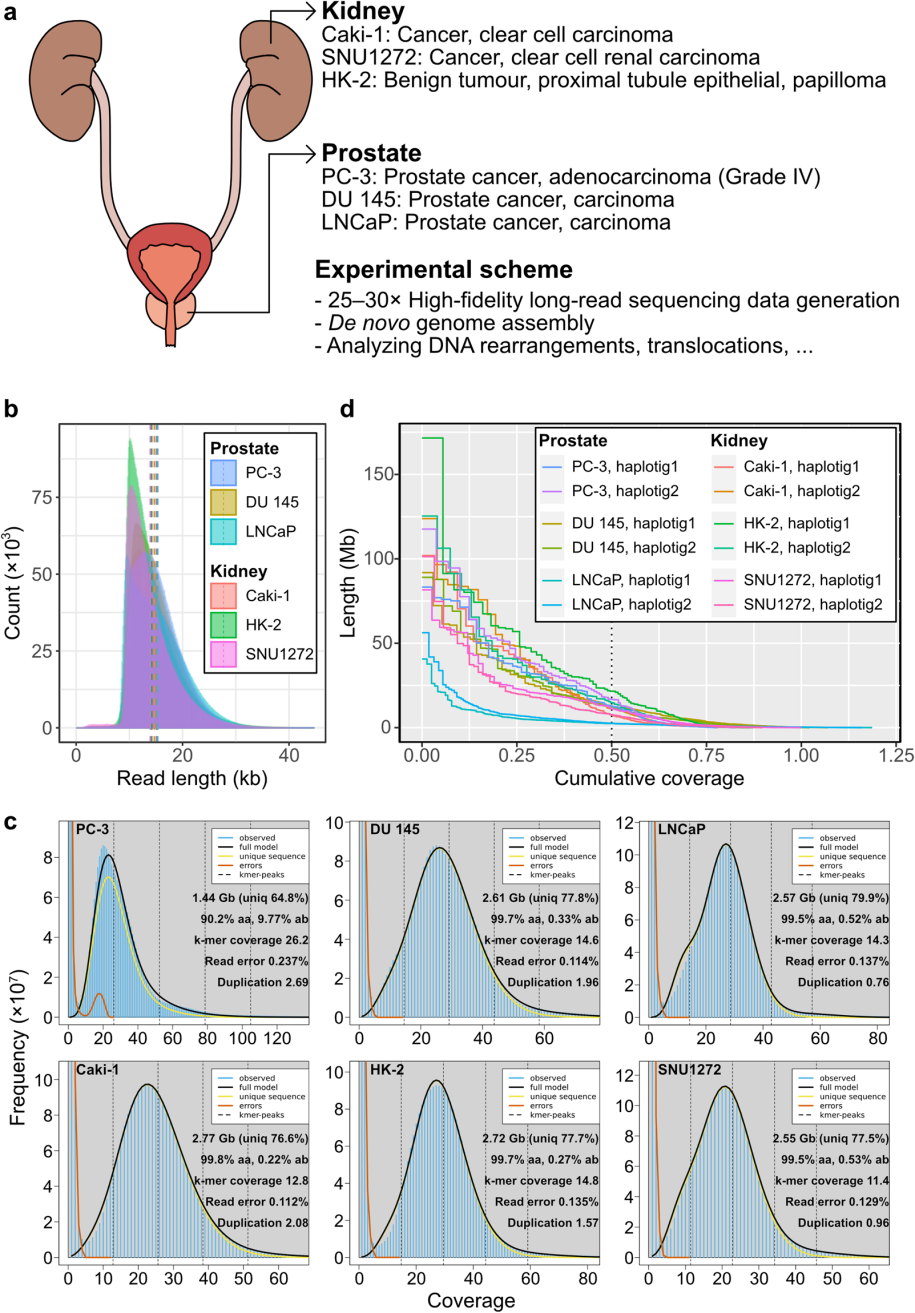
Schematic representation for genitourinary tumour genome assembly (**a**). Cell line information and experimental scheme (**b**). Read length distributions for the six genitourinary tumour cell lines. Each vertical dotted line represents mean read length of each cell line (**c**). *k*-mer coverage plots of the six tumour cell lines. The estimated genome sizes, unique *k*-mer ratios, homozygosity (aa) and heterozygosity (ab) ratios, *k*-mer coverage values, read errors, and duplication rates are displayed on the right side of each panel (**d**). NG plot of the twelve partially phased genome assemblies. T2T-CHM13v2.0 was used for the human genome size calculation. The vertical dotted line represents NG50. Note that the size of some genome assemblies was larger than that of the T2T-CHM13v2.0, resulting in the long right tail of the plot.

**Fig. 2:**
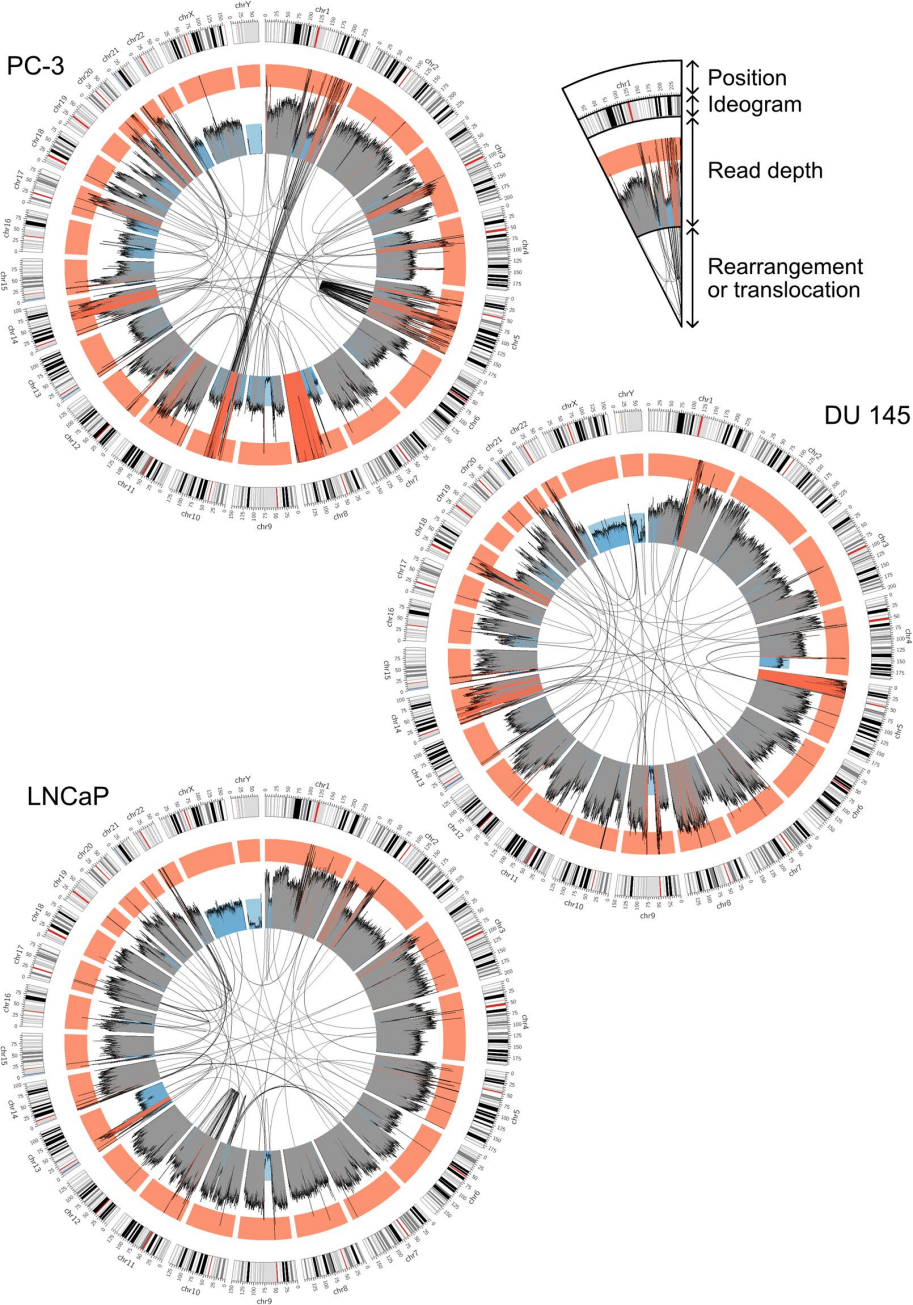
Circos plots of the three prostate cancer genomes. Black lines in the inner circle represent links between any physically linked two loci identified in the genome assembly. Blue, grey, and red histograms in the middle circle represent read-depth distributions of raw HiFi reads mapped to the T2T-CHM13v2.0 genome. Blue represents lower than the half of the mean read depth, red does higher than three halves, and grey does between them. The outer circle represents chromosome ideograms and positions. Red bars represent centromeric regions and black and grey bars do banding patterns.

**Fig. 3:**
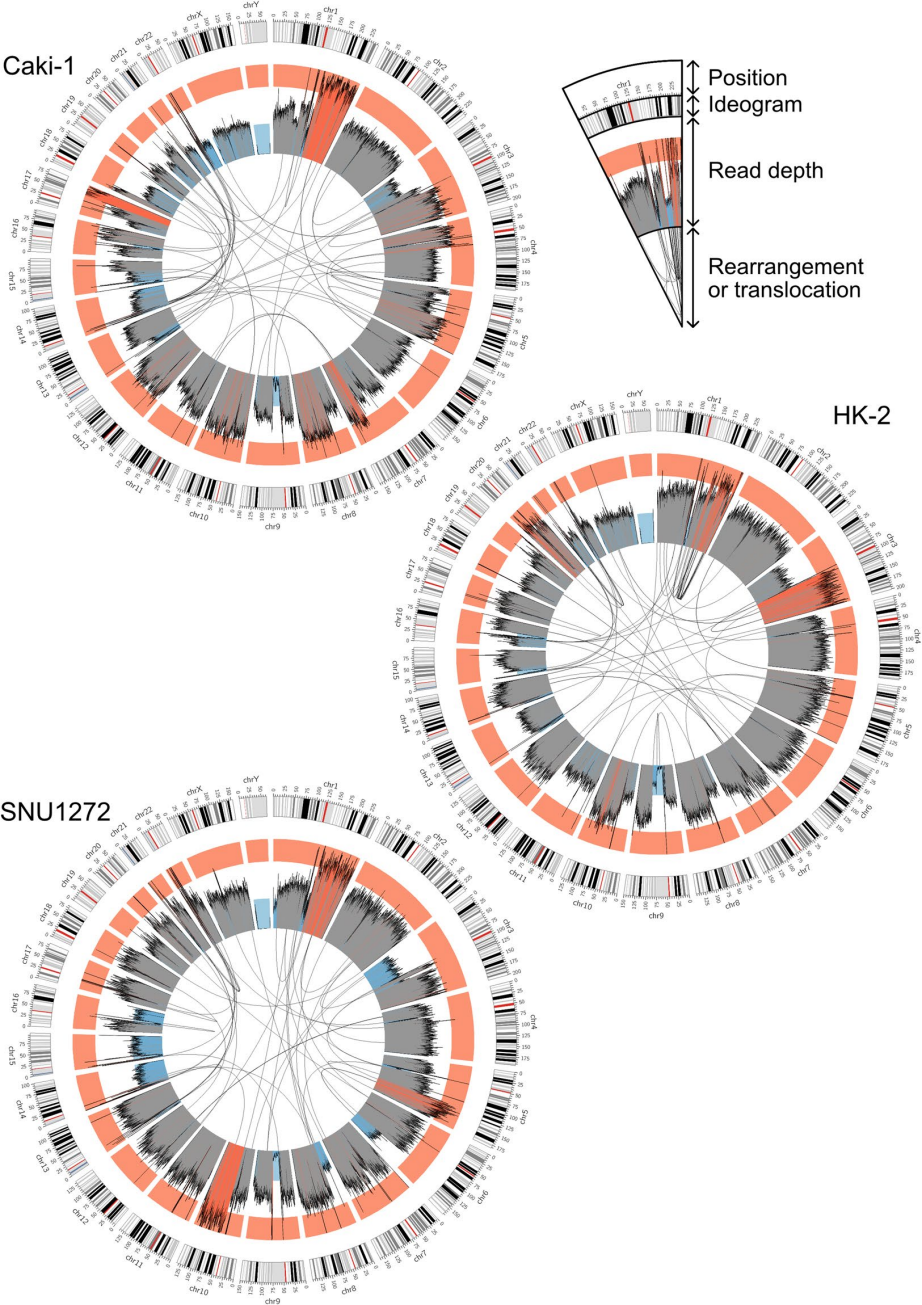
Circos plots of the three kidney tumour genomes. Black lines in the inner circle represent links between any physically linked two loci identified in the genome assembly. Blue, grey, and red histograms in the middle circle represent read-depth distributions of raw HiFi reads mapped to the T2T-CHM13v2.0 genome. Blue represents lower than the half of the mean read depth, red does higher than three halves, and grey does between them. The outer circle represents chromosome ideograms and positions. Red bars represent centromeric regions and black and grey bars do banding patterns.

Here, we provide high-quality genome assemblies five genitourinary cancer and one tumour cell lines (Fig. 1). We utilized high-fidelity (HiFi) long-read sequencing technology of Pacific Biosciences, which provides very accurate and long enough reads (~Q30 and 10–20 kb) in addition to 5-methylcytosine (5mC) information^25–27^. Using HiFi reads and genome assemblies of the cell lines, we assessed large centromeric and telomeric deletions, translocations, and DNA rearrangements that possibly resulted from genome instability (Fig. 2 and Fig. 3). We also established a graph-based draft pan-genome for further mutation analysis, and provided 5-mC maps of the cell lines. Our genomic resources of genitourinary cancers will provide insights into how genome instability creates chromosome-scale mutations in cancers.

## Methods

### Cell line information

In this study, we assessed the following six genitourinary tumour cell lines, all of which were obtained from the Korean Cell Line Bank (KCLB, Seoul, Korea; https://cellbank.snu.ac.kr/). The specific catalogue numbers for each cell line are as follows: KCLB 21435 for PC-3, KCLB 30081 for DU 145, KCLB 21740 for LNCaP, KCLB 30046 for Caki-1, KCLB 22190 for HK-2, and KCLB 01272 for SNU-1272.

- PC-3: The PC-3 cell line originates from bone metastasis and represents an androgen-independent prostate cancer cell line. The genomic characterization of PC-3 cells reveals aneuploidy, with a nearly triploid karyotype consisting of a modal number of 62 chromosomes. Notably, normal Y chromosomes are absent in PC-3 cells.

- DU 145: The DU 145 cell line was established from brain metastasis in a 69-year-old patient diagnosed with prostate cancer. It exhibits a hypotriploid human cell line with chromosome numbers 61 and 62 being most prevalent in 30 metaphase counts. Additionally, higher ploidies are observed in 3% of cells. Several chromosomal abnormalities are present, including t(11q12q), del(11)(q23), 16q + , del(9)(p11), del(1)(p32), and six other marker chromosomes, with the N13 chromosome typically absent. The Y chromosome has undergone translocation to an unidentified chromosomal segment, while the X chromosome is present in a single copy.

- LNCaP: The LNCaP cell line was derived from lymph node metastasis in a Caucasian patient with metastatic prostate cancer. This cell line exhibits a highly aneuploid and hypotetraploid karyotype, with a modal chromosome number of 84 found in 22% of cells.

- Caki-1: The Caki-1 cell line, originating from a metastatic site of skin in a 49-year-old Caucasian male with clear cell renal cell carcinoma, is characterized as aneuploid with chromosome counts falling within the triploid range (modal number = 68; range = 63 to 71). Notably, the Y chromosome is absent. Most normal autosomes, except for chromosomes N9 and N19, are present in two or three copies. Chromosome N9 is recognized as a marker chromosome (M1) that is typically trisomic. Chromosomes N5, N10, and N16 tend to be over-represented compared to other normal chromosomes.

- HK-2: This cell line represents immortalized proximal tubule epithelial cells derived from a normal adult human kidney. It was established through transduction with human papilloma virus (HPV 16) E6/E7 genes. Southern and FISH analyses suggest that the cell line likely originated from a single cell.

- SNU-1272: SNU-1272 is derived from an *in situ* kidney sample and is used for research on clear cell renal cell carcinoma. It was sourced from a 63-year-old female with a genetic profile predominantly reflecting East Asian ancestry.

### PacBio HiFi sequencing and *k*-mer coverage

We cultured the three prostate cancer cell lines, two kidney cancer cell lines, and one benign kidney tumour cell line using Roswell Park Memorial Institute (RPMI) 1640 media (HyClone, Logan, UT, USA) supplemented with 10% fetal bovine serum (FBS) and 1% penicillin and streptomycin (HyClone, Logan, UT, USA). The cells were cultured at 37 °C in a 5% CO_2_ humidified atmosphere. The cells were subcultured once a day for 2–3 days and prepared 10^7^–10^8^ cells for sequencing. DNA extraction and HiFi sequencing using the PacBio Revio system were performed by DNALINK, Korea (http://en.dnalink.com/?redirect=no). Library preparation and sequencing were conducted by DNALINK using the SMRTbell® Prep Kit 3.0, following the manufacturer’s instructions. Briefly, 5 μg of genomic DNA was sheared using the Megaruptor 3 system, followed by end-repair and A-tailing. Short DNA fragments were removed using the BluePippin system with the 0.75% DF Marker S1 High-Pass 6–10 kb vs3 protocol. The SMRTbell adapter was then ligated to the high-molecular-weight DNA, and the resulting HiFi library was loaded onto the Revio Sequencing Plate (25 M ZMWs) for sequencing, with a 24-hour movie time. Produced HiFi read lengths were 14–15 kb and their base quality values were Q31–Q33, in average^28^. A total of 78–92 Gb (5.4M–6.5 M reads; 25–30 × of a human genome size, 3.1 Gb) of HiFi reads were produced for each cell line (Fig. 1 and Table 1).

**Table 1:** Cell line information and read statistics.

Cell line	Accession	Disease	Tissue source	Biological sex	Ploidy	# HiFi reads ( × 10^3^)	HiFi data (Gb)	Mean read length (bp)	Read N50 (bp)	Read quality	Read depth compared to the CHM13 genome	Read depth mapped to the CHM13 genome
PC-3	SRR32197460	Prostate cancer, adeno-carcinoma (Grade IV)	Prostate	Male	Near triploid	5,692	87.3	15,334	15,865	Q31	28.01	27.93
DU 145	SRR32197459	Prostate cancer, carcinoma	Prostate	Male	Hypo-triploid	5,704	86.0	15,075	15,484	Q33	27.59	27.51
LNCaP	SRR32197461	Prostate cancer, carcinoma	Prostate	Male	Hypo-tetra-ploid	5,474	83.5	15,260	16,297	Q31	26.79	26.30
Caki-1	SRR32197462	Kidney cancer, clear cell carcinoma	Kidney	Male	Tri-ploid	5,426	79.7	14,690	15,207	Q32	25.57	24.19
HK-2	SRR32197457	Normal Kidney (proximal tubule epithelial), papilloma	Kidney	Male	Diploid	6,480	92.0	14,203	14,370	Q32	29.51	27.77
SNU1272	SRR32197458	Kidney cancer, clear cell renal carcinoma	Kidney	Female	Near triploid	5,564,617	77.6	13,949	14,098	Q32	24.89	20.70

Long-read genome sequencing data were analysed using the *k*-mer coverage of HiFi reads for each tumour cell line using KMC (version 3.2.4; *kmc -k21 -ci1 -cs10000 reads and kmc_tools transform reads histogram reads.histo -cx10000*) and GenomeScope2 (version 2.0; *genomescope2 -k 21*)^29,30^. The estimated genome sizes ranged from 2.55 to 2.77 Gb and with heterozygosity ratios between 0.22% and 0.57%, except for the PC-3 cell line, which had an estimated genome size of 1.44 Gb and a significantly higher heterozygosity ratio of 9.77%. As the PC-3 cell line contains most of the human chromosomes, this small estimated genome size may suggest that insufficient read depth was obtained for an accurate assessment of *k*-mer coverage for this cell line.

### *De novo* genome assembly

These HiFi reads of each cell line were assembled into partially haplotype-resolved contigs using Hifiasm (version 0.19.5-r587; default option)^17,18^. Output GFA-formatted files were converted into FASTA-formatted files using GNU Awk (version 4.1.4; *awk ‘/^S/{print “* >*“$2;print $3}’*), and their stats were analysed by assembly-stats (version 1.0.1; default option)^31^. Two genome assemblies were generated for each cell line, and these assembly sizes ranged from 2.43 Gb to 3.69 Gb, where two prostate cancer cell lines exhibited larger genome sizes than the other (Fig. 1 and Table 2)^32–43^. Contig N50 lengths were longer than 9 Mb (9.58–29.01 Mb) and largest contig lengths were longer than 50 Mb (81.65–171.62 Mb), implying sufficient contiguity to analyse large structural variants including translocations and rearrangements (Table 2). The only exception was the LNCaP cell line, which exhibited 1.85 and 2.02 Mb of contig N50 lengths and 40.65 and 56.24 Mb of largest contig lengths for its two haplotype-resolved assemblies. Its short contig lengths may result from its extreme genome instability (see below).

**Table 2:** Assembly statistics of the twelve cancer genome assemblies.

Cell line	Disease	Biological sex	Accession	Assembly size (Gb)	Largest contig length (Mb)	Contig N50 length (Mb)	# contigs	Loss of ChrY	# translocations or rearrangements
PC-3	Prostate cancer, adenocarcinoma (Grade IV)	Male	JBDPMX000000000	2.43	83.28	24.78	1,646	Yes	187
			JBDPMY000000000	2.58	117.62	26.26	1,491		
DU 145	Prostate cancer, carcinoma	Male	JBDPNF000000000	3.33	91.85	11.41	4,147	Partial	74
			JBDPNG000000000	3.18	89.03	11.51	3,718		
LNCaP	Prostate cancer, carcinoma	Male	JBDPND000000000	3.69	40.65	1.85	9,515	Yes	116
			JBDPNE000000000	3.61	56.24	2.02	7,941		
Caki-1	Kidney cancer, clear cell carcinoma	Male	JBDPMT000000000	2.70	101.94	13.84	4,333	Yes	55
			JBDPMU000000000	2.58	123.84	19.45	2,326		
HK-2	Normal Kidney (proximal tubule epithelial), papilloma	Male	JBDPMV000000000	2.72	171.62	29.01	3,435	Yes	64
			JBDPMW000000000	2.58	125.37	20.67	1,794		
SNU1272	Kidney cancer, clear cell renal carcinoma	Female	JBDPNB000000000	3.11	81.65	11.28	8,566	Not applicable	52
			JBDPNC000000000	2.80	101.33	9.58	3,164		

It is important to note that our genome assemblies were not fully resolved at the haplotype level. While resolving haplotypes and constructing telomere-to-telomere genome assemblies have been subjects of extensive research, these efforts require additional long-range sequencing information, such as ultra-long-read sequencing data, Hi-C sequencing data, and parental sequencing data^44–52^. This is a significant limitation of our study, as we were unable to obtain such long-range information. However, we have uploaded intermediate graph files from the *de novo* genome assembly to the figshare repository^53^. These files could be utilised in the future to resolve haplotypes, should long-range data become available.

### Analysis of read depth and chromosomal aberration

To analyse all genomic regions using tumour HiFi sequencing data, we utilized the CHM13 genome as a reference, which represents all human autosomes and sex chromosomes without any gap (version GCF_009914755.1_T2T-CHM13v2.0)^44,54^. First, we attempted to identify any visible large insertions and deletions based on raw HiFi read depths (Figs. 2 and 3, inside circles). HiFi raw reads were mapped to the CHM13 using minimap2 (version 2.26-r1175; *minimap2 -a -x map-hifi*), and output mapping files were sorted and indexed using SAMtools (version 1.13; *samtools sort* and *samtools index*)^55–57^. HiFi read depths were calculated for every 200-kb bin using SAMtools (version 1.13; *samtools depth -aa -r*)^57^. Mapped read depths were > 20 × (20.70–27.93 × ) for the CHM13 genome (Table 1).

Impressively, four out of five male tumour cell lines, except for the DU 145, showed that nearly any HiFi reads did not map to the Y chromosome (ChrY), implying that ChrY of the cell lines were lost in these cell lines (Figs. 2 and 3 and Table 2). Even the DU 145 cell line also exhibited very low read depth distribution for its ChrY q-arm. Moreover, our read depth analysis revealed that many, but not all, centromeric and/or telomeric regions were lost or duplicated in their genomes (Figs. 2 and 3). For example, all tumour cell lines exhibited very low read depths in their centromeres of Chr9 and Chr16, in addition to the centromeric and telomeric regions in Chr15. It indicates that these regions were lost in all chromosomes in these six tumour cell lines. It may result from the breakage-fusion-bridge (BFB) cycle.

Second, we analysed translocations and chromosome rearrangements in our tumour genome assemblies. Our genome assemblies were aligned to the CHM13 genome using Winnowmap2 (version 2.03; *meryl count k = 19*, *meryl print greater-than distinct = 0.9998*, and *winnowmap -W -ax asm20–cs -r2k*), and output alignment files were sorted and indexed using SAMtools (version 1.7; *samtools sort* and *samtools index*)^57,58^. Translocation and rearrangement loci were identified using SVIM-asm (version 1.0.2; *svim-asm haploid*)^59^. Translocations between different chromosomes or rearrangements between two loci at 10-Mb distance in a chromosome were further analysed. Prostate cancer cell lines exhibited many more translocation or rearrangement loci than kidney tumour cell lines (74–187 vs. 52–64 for each cell line) (Figs. 2 and 3 and Table 2).

These translocation and rearrangement loci identified by tumour genome assemblies further supported read-depth analysis results: Most of the centromeric and telomeric copy-number variations may result from the BFB cycle (Figs. 2 and 3 and Table 3). For example, deleted regions near the Chr9 centromere identified by read depths were translocation loci. Deleted regions in Chr15 and Chr16 were also identified as translocation loci in tumour genome assembly results, except for the Chr16 centromeric deletion in SNU1272, which was a rearrangement locus. Since these loci mostly exhibited close positions, but were not the same, they may not be errors in sequencing, assembly, or translocation analysis (Figs. 2 and 3 and Table 3). In addition, LNCaP and PC-3 exhibited extreme translocation/rearrangement events in their genomes (Fig. 2), which possibly causes low contiguity of LNCaP (Table 2).

**Table 3:** Examples of translocation and rearrangement loci in Chr9, Chr15, and Chr16.

Cell line	Start chromosome	Start position	Target chromosome	Target position	Type
DU145	chr9	48,503,323	chr3	129,306,048	Translocation
LNCaP	chr9	48,544,002	chr5	49,608,352	Translocation
SNU1272	chr9	48,879,598	chr15	647,827	Translocation
Caki-1	chr9	67,124,376	chr3	77,530,190	Translocation
DU145	chr9	79,076,161	chr14	6,962,077	Translocation
PC-3	chr9	79,076,161	chr21	9,430,108	Translocation
HK-2	chr9	93,707,531	chr8	146,229,729	Translocation
SNU1272	chr15	2,397,559	chr22	41,218,685	Translocation
LNCaP	chr15	5,440,385	chr13	13,824,863	Translocation
DU145	chr15	11,891,498	chr20	25,582,482	Translocation
Caki-1	chr15	13,718,418	chr11	68,268,333	Translocation
HK-2	chr15	14,474,659	chr21	8,601,231	Translocation
PC-3	chr15	14,485,791	chr22	12,210,437	Translocation
DU_145	chr16	30,315,566	chr19	15,378,827	Translocation
HK-2	chr16	34,319,582	chr8	44,590,780	Translocation
PC-3	chr16	35,524,960	chr20	33,611,114	Translocation
LNCaP	chr16	38,683,949	chr9	66,622,680	Translocation
SNU1272	chr16	40,751,203	chr16	50,753,822	Rearrangement
Caki-1	chr16	50,533,465	chr19	25,980,094	Translocation

### Graph-based pan-genome analysis for tumour genome assemblies

Genetic variants in our 12 tumour genome assemblies were analysed for each cell line based on their respective genome graphs constructed using the Minigraph-Cactus pan-genome pipeline^60,61^ (version 2.6.4; *cactus-pangenome jobStorePath sequenceFilePath.tsv --reference CHM13 --vcf --giraffe–gfa --gbz*). We additionally utilised the raw HiFi read mapping files from our read depth analysis to generate mapping-based variant call sets, using DeepVariant for SNPs (version 1.2.0; *run_deepvariant --model_type PACBIO --ref CHM13 --reads rawHiFi.bam*) and Sniffles for SVs (version 2.0.7; *sniffles --input rawHiFi.bam --reference CHM13*)^62,63^.

A total of 3.9M–6.6 M variants were detected for each cell line using the pangenome pipeline. Of these, only 123–401 loci were obviously mis-called, as the number of alleles exceeded the total number of input assemblies of each cell line that is, 2. However, the number of mis-called loci was significantly higher for DU 145 and LNCaP, with 6,247 and 27,770 mis-calls, respectively (Table 4). Among the remaining variants, 3.1M–4.4 M were single-nucleotide polymorphisms (SNPs), and 16K–23 K were structural variants (SVs; variants size ≥ 50 bp) (Table 4). We then assessed whether these SNPs and SVs were supported by mapping-based variant call sets. We found that 91%–94% of assembly-based SNPs and 37%–45% of assembly-based SVs were shared with the mapping-based call sets (Table 4). Additionally, we used other assembly-based SV call sets identified using SVIM-asm and found that 87%–92% of the supported SVs for both Sniffles (mapping-based) and SVIM-asm (assembly-based) were shared (Table 4). These data are available in the EVA and figshare databases^53,64^.

**Table 4:** Variant summary statistics of a cancer pan-genome.

Sample	Total variant	Loci with ≥ 3 alleles	Total SNP	Valid SNP (DeepVariant)	Total SV	Valid SV (Sniffles)	Valid SV (SVIM-asm)	Valid SV (Both)	Valid SV (Both), germline	Valid SV (Both) somatic
PC-3	3,921,883	153	3,067,055	2,875,966	16,426	7,461	7,396	6,815	4,439	2,376
DU 145	5,603,293	6,247	3,951,173	3,604,767	20,167	8,204	8,254	7,373	4,801	2,572
LNCaP	6,567,787	27,779	4,441,117	4,049,702	23,297	8,540	8,820	7,692	5,020	2,672
Caki-1	4,496,625	205	3,496,096	3,290,017	18,709	8,058	8,023	7,297	4,729	2,568
HK-2	4,244,617	123	3,317,251	3,134,620	17,290	7,788	7,786	7,106	4,604	2,502
SNU1272	4,781,782	401	3,720,611	3,490,157	19,273	8,345	8,329	7,545	4,857	2,688

It is noteworthy that these variants may include common variants found in the human population rather than being tumour-specific. Genome assemblies of matched normal cell lines would be beneficial for distinguishing non-tumour-specific variants^65,66^, but we were unable to obtain such lines for the six genitourinary tumour cell lines used in this study. Due to these limitations, we aimed to distinguish germline and somatic SVs by utilizing SVs reported from long-read sequencing data in population studies. Through this approach, we identified that approximately 65% of the variants were germline, while 35% were potential somatic variants (Table 4)^67^. We hope that future studies can extract both tumour and normal somatic cells from the same individual to more thoroughly separate tumour-specific SNPs and SVs.

### 5-Methylcytosine sites in tumour cell lines

We annotated 5mC by mapping BAM-formatted HiFi reads that contain the base modification tag information to the CHM13 genome using pbmm2 (version v1.12.0; *pbmm2 align chm13.fa cancer_HiFi.bam output.bam --preset HIFI --sort*)^68^. The output mapping files were used to identify 5mC sites using pb-CpG-tools (version 2.3.1; *aligned_bam_to_cpg_scores --model pileup_calling_model.v1.tflite*)^69^. All tumour cell lines exhibited ~30 million potential 5mC sites, but the number of filtered 5mC sites ( ≥90% probability) ranged from 10 M to 18 M (Table 5). These filtered 5mC sites covered 0.32%–0.59% of the CHM13 genome and 14.68%–27.05% of its CpG sites. Prostate cancer cell lines exhibited slightly lower coverage than kidney tumour cell lines, as PC-3 and LNCaP cell lines, which showed higher numbers of translocations and rearrangements, exhibited the two lowest numbers of 5mC sites.

**Table 5:** Summary statistics for 5-methylcytosine sites.

Cell line	Disease	Number of all 5mC sites	Number of 5mC sites, ≥ 90% probability	5mC ratio to the genome size ( ≥ 90% probability)	5mC ratio to the CpG sites( ≥ 90% probability)
PC-3	Prostate cancer, adenocarcinoma (Grade IV)	29,917,642	10,914,326	0.35	16.10
DU 145	Prostate cancer, carcinoma	30,711,231	17,156,567	0.55	25.31
LNCaP	Prostate cancer, carcinoma	30,590,242	9,948,928	0.32	14.68
Caki-1	Kidney cancer, clear cell carcinoma	30,117,431	14,617,076	0.47	21.56
HK-2	Normal Kidney (proximal tubule epithelial), papilloma	29,931,210	18,340,451	0.59	27.05
SNU1272	Kidney cancer, clear cell renal carcinoma	29,782,354	13,701,819	0.44	20.21

**Fig. 4:**
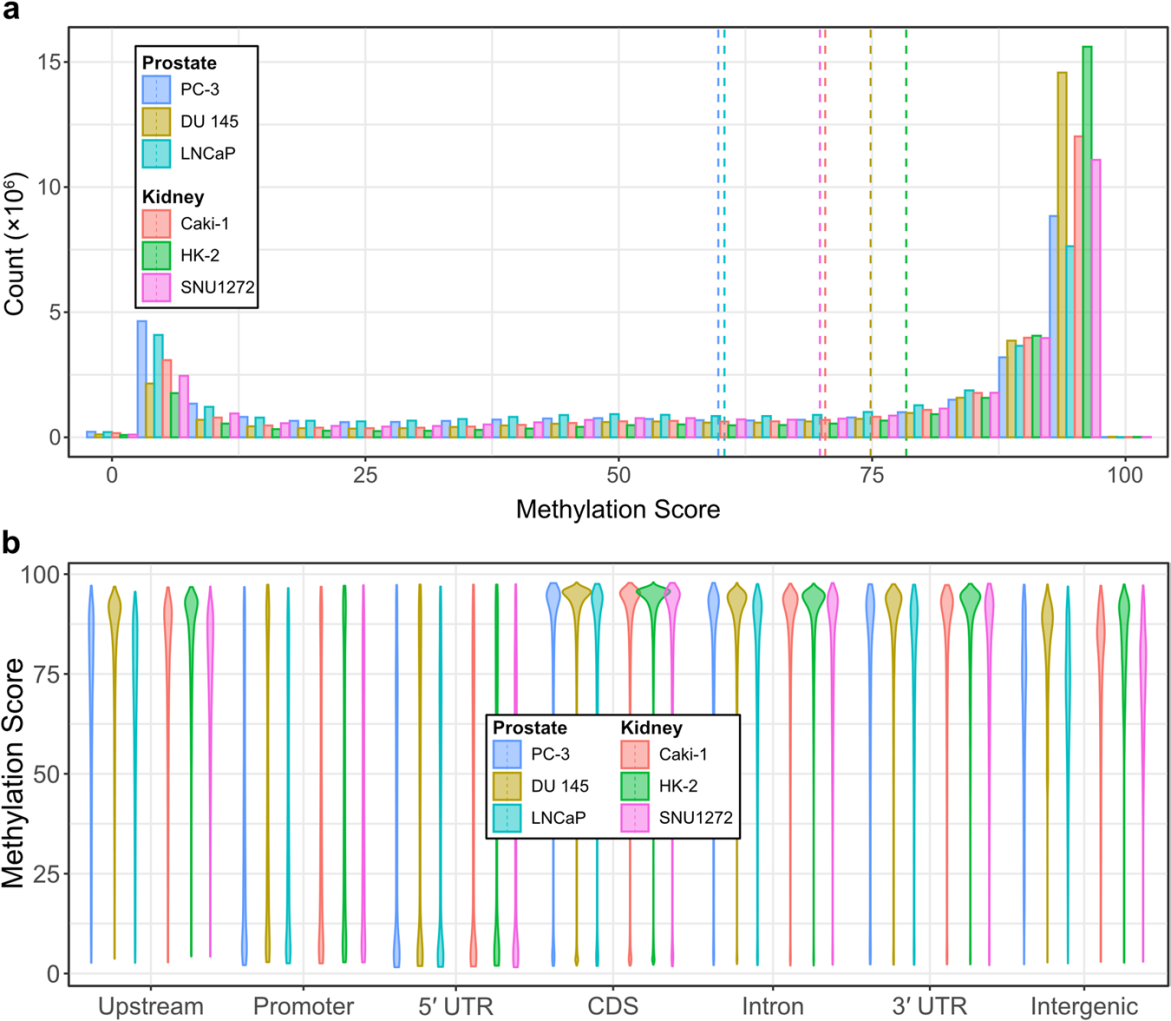
DNA methylation profiles of six genitourinary tumour cell lines (**a**). Count distribution of methylation scores calculated using pb-CpG-tools (**b**). Methylation distribution across various genomic regions, including upstream regions (1–5 kb from the transcription start site), promoter regions (0–1 kb from the transcription start site), 5′ untranslated regions (UTRs), coding sequences (CDS), intronic regions, 3′ UTRs, and intergenic regions.

These methylated sites were primarily depleted in the 5′ untranslated regions (5′ UTRs) and promoter regions (Fig. 4). The distribution of methylation scores showed that the majority of the genome is highly methylated, with DU 145 and HK-2 displaying a significantly higher number of highly methylated sites compared to the other tumour cell lines (Fig. 4a). Methylation patterns were also analysed across genic and non-genic regions. All cell lines exhibited lower levels of methylation in the 5′ UTRs and promoter regions (Fig. 4b). Although DU 145 and HK-2 followed similar overall patterns to the other cell lines, they showed elevated methylation scores, particularly in upstream and intergenic regions (Fig. 4b). Future studies could investigate whether these two cell lines are more effective at inhibiting random transcription in non-coding regions compared to the other cell lines.

## Data Records

Our genome assemblies and raw PacBio reads in FASTQ format and BAM format were submitted to the NCBI BioProject database (https://www.ncbi.nlm.nih.gov/bioproject) under the accession number PRJNA1035301. All reads are available under SRP470038^28^, and genome assemblies are accessible as follows: GCA_040939165.1^34^ and GCA_040939315.1^37^ for PC-3; GCA_040939345.1^40^ and GCA_040939335.1^39^ for DU 145; GCA_040939435.1^43^ and GCA_040939415.1^42^ for LNCaP; GCA_040939145.1^32^ and GCA_040939155.1^33^ for Caki-1; GCA_040939185.1^36^ and GCA_040939175.1^35^ for HK-2; and GCA_040939325.1^38^ and GCA_040939355.1^41^ for SNU1272. Genome assemblies, read depth summary, pan-genome validation, and 5-methylcytosine output files were also uploaded to figshare (10.6084/m9.figshare.27021865)^53^.

## Technical Validation

Extracted DNA exhibited high purity and high-molecular-weight (Nanodrop: ~1.8 for 260/280 and ~2.2 for 260/230; Agilent Femto Pulser: 18,431–41,296 bp of Femto size). We obtained ~25× HiFi sequencing data of which read quality exceeded Q30 (Q31–Q33) and mean read lengths were ~14 kb (13,949–15,260 bp). Assembly contiguity was analysed by their contig lengths using assembly-stats as described in the Methods section. Contig N50 was 9.58–29.01 Mb and largest contig N50 was 81.65–171.62 Mb, except for the LNCaP cell line that exhibited ~2 Mb of contig N50 and ~40 Mb of largest contig N50.

## Data Availability

All programs and pipelines were executed following their official manuals or help pages. Version and parameter information that we used for our analysis have been described in the Methods section. No custom scripts were used.
